# Summary of the Therapeutic Options for Patients with Dry and Neovascular AMD

**DOI:** 10.3390/jcm13144227

**Published:** 2024-07-19

**Authors:** Dorota Śpiewak, Łukasz Drzyzga, Mariola Dorecka, Dorota Wyględowska-Promieńska

**Affiliations:** 1 Department of Ophthalmology, Prof. K. Gibiński University Clinical Center, Medical University of Silesia, 40-514 Katowice, Poland; 2 Clinical Ophthalmology Center Okolux, 40-754 Katowice, Poland; 3 Department of Ophthalmology, Faculty of Medical Sciences in Katowice, Medical University of Silesia, 40-287 Katowice, Poland

**Keywords:** age-related macular degeneration, complement system, anti-VEGF, gene therapy, drug delivery systems

## Abstract

Age-related macular degeneration (AMD) is the leading cause of irreversible blindness worldwide and a severe medical and social problem. The steadily increasing number of patients is related to the aging of the population. So far, many factors affecting the development of AMD have been identified, which can be divided into non-modifiable, including genetic factors, age, and sex, and modifiable or environmental factors, such as smoking, poor diet, and hypertension. Early stages of age-related macular degeneration are characterized by fundus drusen and abnormalities in the retinal pigment epithelium. In late stages, geographic atrophy and choroidal neovascularization (CNV) are observed. The treatment of AMD, especially its advanced forms, is very challenging. Intensive research has made it possible to treat advanced stages of the dry form of AMD with pegcetacoplan and avacincaptad pegol, new drugs approved for use in the US. Pegcetacoplan targets the C3 and avacincaptad pegol targets the C5, the pivotal proteins of the complement cascade. The drugs are administered by intravitreal injection. The gold standard for neovascular AMD (nAMD) consists of intravitreal injections of anti-vascular endothelial growth factor (anti-VEGF) drugs such as bevacizumab, ranibizumab, aflibercept, brolucizumab, and faricimab. Treatment can be administered according to the fixed, pro-re-nata, and treat-and-extend regimens. The latter seems to have the best effect on improving visual acuity (VA) and the maximum therapeutic benefit. The search continues for the best ways to deliver intravitreal drugs. Current methods include sustained-release implants and hydrogel platforms for drug release, while the most promising future pathways for treating dry and nAMD are stem cell and gene therapy.

## 1. Introduction

Age-related macular degeneration (AMD) is the leading cause of severe vision loss, often leading to blindness in people over 60 years of age worldwide. AMD is a progressive disease in which decreased visual acuity (VA) affects central vision regardless of distance. Fine details become difficult or impossible to see, while peripheral vision remains normal [1]. In terms of severity, there are three forms of AMD. The early form is characterized by changes in the retinal pigment epithelium (RPE) and/or hard small drusen. The intermediate form is characterized by soft large drusen and/or geographic atrophy (GA) of the RPE sparing the fovea. The late form is characterized by GA with foveal involvement and/or choroidal neovascularization (CNV) [2]. In addition to the division according to lesion severity, we distinguish between dry and neovascular forms of AMD. Degenerative changes are typical of both forms; their exact etiology remains unclear. The pathogenesis of AMD involves lipofuscinogenesis, drusenogenesis, local inflammation, and neoangiogenesis [3]. AMD is a complex multifactorial disease associated with genetic and environmental risk factors. Age, genetic factors, sex, the protective brown color of the irises, and the adverse effect of hyperopia are non-modifiable risk factors for the development of AMD. Age shows the highest correlation with the likelihood of developing AMD, with odds ratios (ORs) ranging from 1 at age 55–69 to 4.42–8.70 at age 70–79 and 18.8–32.3 at age 80 to 86 [2]. A large number of genes may be responsible for the development of AMD. Polymorphisms that modify susceptibility to the disease have been found in at least 37 genes in neovascular (nAMD) of the Single Nucleotide Polymorphism database (dbSNP), listed in Table 1.

The main risk factor for AMD is the 402H variant of the complement factor H (CFH) gene. The diseased risk is 2–4 times increased in heterozygotes and 4–7 times in homozygotes. The CFH gene increases the risk of dry AMD, drusen, geographic atrophy, and nAMD equally. A strong association has been shown between all stages of AMD and the genetic variation in age-related maculopathy susceptibility (ARMS2). In patients homozygous for the risk allele, the lack of ARMS2 synthesis is the cause of AMD. ARMS2 is also essential for the proper functioning of the extracellular matrix. The Pigment Epithelium-Derived Factor (PEDF) gene belongs to the serine protease inhibitor gene family. Mutations within this gene can result in various retinal diseases, including AMD and polypoidal choroidal vasculopathy (PCV). PEDF is a 50 kDa protein first isolated from a conditioned medium of human RPE cells as an inducer of neuronal differentiation of cultured Y79 retinoblastoma cells. PEDF is a multifunctional protein with anti-angiogenic, antioxidant, neuroprotective, and neurotrophic effects. The clinical use of PEDF may significantly improve the future treatment of CNV by reducing vascular leakage and protecting retinal photoreceptors [4,5,6,7]. Race and ethnicity may also play an important role. Caucasians have a higher risk of developing AMD compared to black people or white Hispanic people. Significant modifiable risk factors for the development of AMD are smoking (OR: 2.39–4.22), overweight (OR 1.06–1.35), hypercholesterolemia, hypertension (OR 1.02–1.48), previous cataract surgery (OR: 1.59), and a family history of AMD (3.95–6.98) [2].

The pathogenesis of AMD has yet to be fully understood. During AMD, degenerative changes involve the outer layers of the retina, such as the photoreceptors, RPE, Bruch’s membrane, and choriocapillaris. Hyaline drusen form in Bruch’s membrane and the RPE; at a later stage, lipofuscin deposits form and photoreceptor atrophy, as well as CNV, leads to scar formation. Drusen are usually the first ophthalmoscopic manifestation of AMD and appear before any noticeable deterioration in visual function. Deposition of drusen in Bruch’s membrane leads to thickening and reduced permeability, impairing both the transport of nutrients to the retina and the transfer of metabolic products to the choroid; it is accompanied by choroidal vascular thinning. In combination with neurodegenerative changes within the photoreceptor-RPE complex, drusen cause RPE abnormalities, including hypo- or hyperpigmentation, in the early and intermediate stages of the disease. This combination of factors leads to impaired RPE and photoreceptor function [8].

The pathological changes develop in the same retinal layers in both dry AMD and nAMD; dry AMD is believed to be the precursor to nAMD. Reactive oxygen species and free radicals generated by metabolism expose RPE cells to oxidative stress, disrupting lipid metabolism, extracellular matrix remodeling, and macrophage recruitment. Oxidative stress can result in the accumulation of drusen, which mechanically damage RPE cells and cause inflammatory processes by interfering with the transport of nutrients and oxygen from Bruch’s membrane to the photoreceptors. This results in RPE atrophy and induction of irreversible neovascularization processes. Hyaline and lipofuscin drusen complexes cause the accumulation of the complement component C5a and IgG antibodies at this site, interfering with the recruitment of macrophages necessary for the phagocytosis of photoreceptor outer segments. In addition, C5a and IgG stimulate the choroid to secrete the chemokine CCL2 (MCP-1), which promotes further accumulation of C5a and IgG, resulting in the expression of VEGF by RPE cells [9,10], as well as migration and accumulation of monocytes (MCP) and interleukin-8 (IL-8). These processes lead to an imbalance between pro-angiogenic and anti-angiogenic factors, such as PEDF. Retinal hypoxia triggers the production of VEGF and pro-inflammatory cytokines, which initiate neovascularization processes in the retina through the proliferation and migration of choriocapillaris endothelial cells. Macrophages migrate from the choriocapillaris along the outer layer of Bruch’s membrane and accumulate around sites of vascular growth. Macrophage activation upregulates the tumor necrosis factor (TNF) expression, stimulating the RPE to produce IL-8, MCP, and VEGF. TNF-α also stimulates the expression of α-3 and α-5 integrins within the RPE, triggering cell migration via tyrosine kinase. New vessels, still lacking structural integrity, penetrate Bruch’s membrane due to protease-mediated activation of VEGF and cytokines. The newly formed capillaries differentiate into arterioles and venules, growing through the RPE into the subretinal and intraretinal space. A key role at this stage belongs to mitochondrial matrix metalloproteinase (MMP), which allows CNVs to pass through individual tissues [11].

The pathogenesis of the nAMD involves the recruitment of immune cells to the damaged macula and the secretion of pro-inflammatory and pro-angiogenic cytokines, particularly the factor VEGF. This factor stimulates the proliferation and migration of endothelial cells and leads to angiogenesis and increased vascular permeability. Fluid from newly formed pathological blood vessels penetrates beyond the vascular bed, causing damage to photoreceptors, which contributes to decreased VA. The resulting neovascular membrane leads to macula fibrosis, atrophy, and irreversible loss of central vision [12].

Angiogenesis is the formation of blood vessels from existing vessels due to an imbalance between pro- and anti-angiogenic factors. Proangiogenic factors include VEGF, nitric oxide (NO), integrins (α5β1, αvβ3, and αvβ5), transforming growth factor-beta 1 (TGF-beta 1), acid fibroblast growth factor (aFGF), basic fibroblast growth factor (bFGF), hepatocyte growth factor (HGF), insulin-like growth factor I (IGF-1), platelet-derived growth factor (PDGF), hypoxia-induced IL-8 and IL-1, prostaglandins (PGE 1, PGE 2, and PGF), erythropoietin, histamine, bradykinin, and TNF-α [13]. 

TNF, also referred to as TNFα, comprises three identical polypeptide chains joined non-covalently (homotrimers). The TNF precursor is synthesized as a transmembrane protein (pro-TNF). After processing by TNF-α-converting enzyme, the soluble form of TNF-α is released [14]. TNF-α plays an essential role in the pathophysiology of AMD. It is a cytokine involved in inflammation-related neoangiogenesis. TNF-α is generated by macrophages and T-lymphocytes; it increases VEGF expression in RPE and choroidal fibroblasts. TNF-α additionally stimulates monocyte adhesion and increases the expression of granulocyte-macrophage colony-stimulating factor [15]. These processes lead to the formation of abnormal blood vessels in the retina, which is characteristic of nAMD. TNF-α exacerbates oxidative stress in the retina, which can induce damage and apoptosis of RPE cells and photoreceptors, thus contributing to the progression of nAMD. TNF-α modulates the expression of various apoptotic factors in RPE cells and other proinflammatory cytokines such as IL-8. The nuclear factor kappa-light-chain-enhancer of activated B cells (NF-κB) is a protein complex that acts as a transcription factor found in almost all animal cells, taking part in the cell’s response to stress stimuli. Through its effect on the NF-κB pathway, TNF-α redirects cells to transition from a pro-inflammatory response to a state where cellular defense mechanisms can no longer cope with the challenge, resulting in increased cellular damage. It has also been shown that, through induction of complement factor B (CFB), TNF-α influences an alternative pathway in the development of AMD. In clinical trials, intravitreal injections of infliximab, a monoclonal antibody against TNFα, have not significantly improved VA or retinal structure in patients who do not respond to anti-vascular endothelial growth factor (anti-VEGF) therapy. In addition, intravitreal infliximab can induce severe intraocular inflammatory reactions [16,17]. Despite inconsistent findings of clinical trials with anti-TNFα drugs conducted to date, targeting TNF with various therapeutic strategies is still considered a promising treatment for AMD, potentially slowing disease progression. The role of anti-TNFα in AMD is still incompletely understood; the implications of anti-TNFα as a therapeutic agent in treating AMD are still under investigation [17].

Subretinal neovascularization is believed to be triggered by local inflammation and immune reactivity. The recruitment of immune cells to areas of macular damage and atrophy causes the secretion of pro-inflammatory and pro-angiogenic cytokines, including VEGF [13]. The VEGF subfamily is functionally diverse and consists of VEGF-A, VEGF-B, VEGF-C, VEGF-D, VEGF-E, and placental growth factor (PIGF). VEGF-A is involved in angiogenesis, vasodilation, and nitric oxide release; it enhances the chemotaxis of macrophages and granulocytes. VEGF-B participates in neovascularization, occurring during embryonic development and in the progression of tumor growth. VEGF-C enhances vascular permeability and is involved in lymphangiogenesis. VEGF-D is a protein that affects the remodeling of blood and lymphatic vessels and significantly negatively affects disease processes in the body. VEGF exists in at least six isoforms in the human body, i.e., VEGF121, VEGF145, VEGF165, VEGF183, VEGF189, and VEGF206. All VEGF family proteins stimulate a cellular response by binding to tyrosine kinase receptors (VEGFRs) on the cell surface, resulting in their dimerization and activation by trans-phosphorylation. VEGF-A binds to VEGFR-1 (FLT-1) and VEGFR-2 (KDR/FLT-1) receptors. VEGFR-2 mediates almost all known cellular responses to VEGF. The function of VEGFR-1 has yet to be established but it is thought to modulate the signaling action of VEGFR-2. The third receptor, VEGFR-3, does not bind VEGF-A. Its ligands are VEGFR-C and VEGFR-D and it mediates lymphangiogenesis [18]. Angiogenic factors act on the endothelium of blood vessels, which are usually resistant to neovascular stimuli. In particular, VEGF-A and placental growth factor (PLGF) have been shown to activate inactive endothelial cells and promote their proliferation, migration, and increased permeability [13]. The endothelial Tie-1 and Tie-2 receptor tyrosine kinases are involved in the angiogenesis pathway in the retina [19]. Angiopoetin-1 (Ang-1) and angiopoetin-2 (Ang-2) bind to the Tie-2 receptor and are essential regulators of vascular stability. The functions of Ang-1 and Ang-2 in angiogenesis are essentially opposite. Angiopoietins 1 and 2 are ligands for the receptor tyrosine kinase Tie2, found mainly in vascular endothelial cells. Ang-1 activates the Tie-2 receptor by inducing receptor tyrosine phosphorylation. Ang-2 can compete with Ang-1 in binding to the receptor, inhibiting Ang-1-induced tyrosine phosphorylation. Ang-1 acts as an endothelial stabilizer. The action of Ang-2 appears to be VEGF-dependent. In the absence of VEGF, Ang-2 promotes vessel regression; the presence of VEGF induces angiogenesis [20].

In nAMD, neovascularization can begin in the outer retina or choroid. There are three types of neovascularization: Type 1 macular neovascular membranes (MNV), in which vascular ingrowth starts from the choroid into and beneath the RPE; type 2 MNV, in which neovascularization starts from the choroid, penetrates Bruch’s membrane and the RPE layer, and then proliferates into the subretinal space; and type 3 MNV, in which neovascularization originates from the retinal circulation, usually in the deep capillary plexus, and grows toward the outer retina and choroid. 

PCV, a subclassification of type 1 neovascularization, is considered the specific phenotype of nAMD. The disease occurs more frequently in African and Asian populations, with a prevalence of 22–62% [13]. It usually involves pigment epithelial detachment, exudation, bleeding, and subretinal fibrosis [21].

## 2. Treatment of Dry AMD

### 2.1. General Information

**Figure 1:**
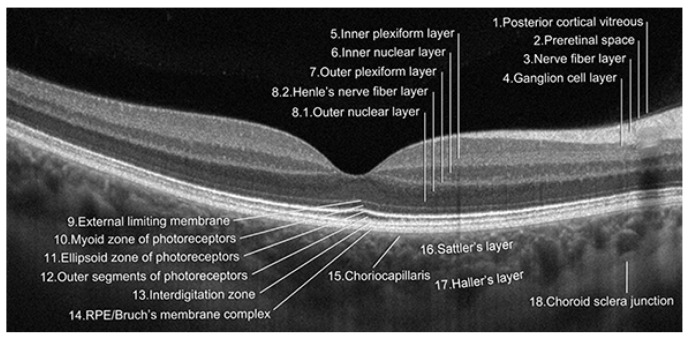
Nomenclature for normal anatomy landmarks seen in OCT (proposed and adopted by the International Nomenclature for Optical Coherence Tomography Panel).

**Figure 2:**
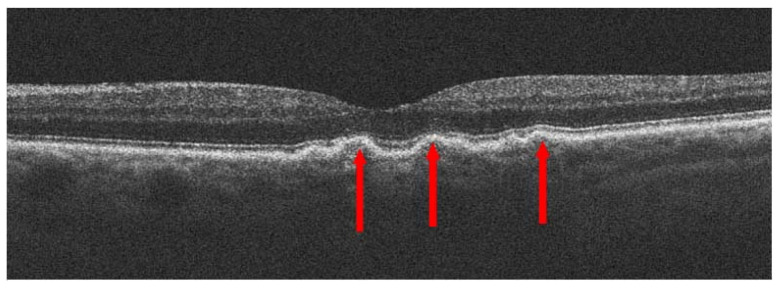
Dry age-related macular degeneration (dry AMD); red arrows point to drusen.

**Figure 3:**
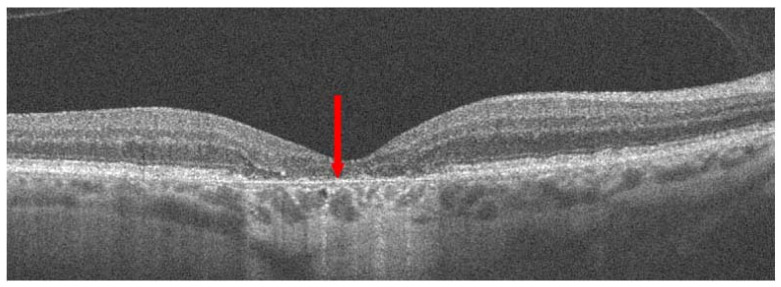
Dry AMD; red arrow indicates geographic atrophy (GA).

**Figure 4:**
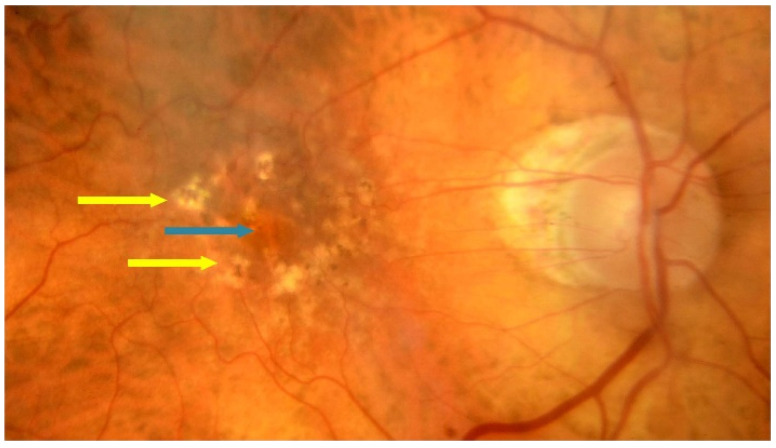
Dry AMD–fundus; yellow arrows point to hard drusen and blue arrow shows atrophy.

Dry AMD accounts for nearly 80–85% of all AMD diagnoses. Central retinal atrophy spreads over the years within the macula but never affects the retina’s periphery. Close-up vision is the most impaired, at first making it impossible to see a few letters in a word and then whole words. Figure 1 shows an optical coherence tomography (OCT) scan of normal retinal structure for comparison with the described pathologies occurring in the retina during AMD. The characteristic clinical manifestation of dry AMD is retinal deposits, otherwise referred to as drusen, shown in Figure 2. RPE changes, including pigment clumping or abnormal autofluorescence, may also be early clinical signs. The type and amount of drusen determine the early, intermediate, and late stages of dry AMD but the deterioration in VA is not necessarily due to retinal deposits. The main cause of severe vision loss in AMD is progression to geographic atrophy [22], shown in Figure 3. Dry AMD fundus is presented in Figure 4.

**Figure 5:**
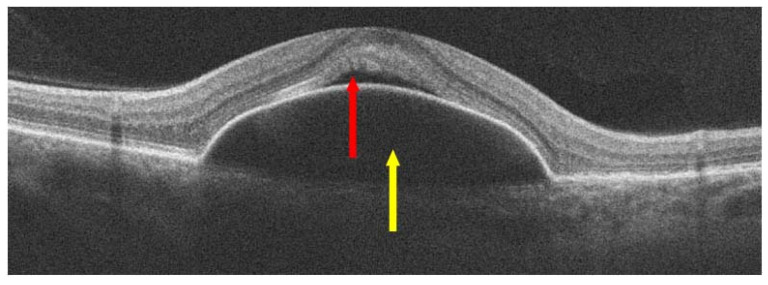
Non-neovascular AMD; yellow arrow shows pigment epithelial detachment (PED) and red arrow shows subretinal fluid (SRF) at the apex of the lesion.

Non-neovascular AMD with subretinal fluid (SRF) is an important clinical entity that should be recognized to avoid anti-VEGF therapy, which is unnecessary in these patients. SRF may be due to drusen or drusenoid pigment epithelial detachment (PED) that is not associated with macular neovascularization but with retinal pigment epithelial decompensation or dysfunction [23]. Figure 5 shows an example OCT scan.

Lifestyle modification and dietary supplementation have been shown to be of benefit to patients with dry AMD. The treatment of early AMD should include health promotion by emphasizing a healthier lifestyle, including diet, exercise, and smoking cessation. Nutrition education should promote the consumption of foods containing xanthophyll carotenoids, which play a significant role in maintaining macular integrity and may further help increase macular pigment optical density (MPOD). These products include egg yolk, spinach, kale, savoy cabbage, and colored vegetables such as peppers [24]. In addition, vitamin and antioxidant supplements, known as the AREDS2 formula, are also used, which can reduce the risk of vision loss. The large AREDS study and the subsequent AREDS2 study showed that daily intake of specific vitamins and minerals can slow the development of dry AMD. Vitamin C (500 mg/d) is a powerful antioxidant, protecting the body from free radicals that cause oxidative stress; its deficiency can cause lipofuscin accumulation and loss of photoreceptors. Vitamin E (400 IU/d) is also a powerful antioxidant. Lutein (10 mg/d) and zeaxanthin (2 mg/d) are xanthophyll carotenoids that increase MPOD and improve visual functions such as contrast sensitivity and glare tolerance. Copper (2 mg/d) protects against oxidative stress. Zinc (80 mg/d) is a cofactor for metabolically active enzymes and plays a crucial role in maintaining macular stability and neuronal structure and function [25,26].

New research on AMD treatment focuses on preventing the progression of degeneration and repopulating the atrophic macula. The development of dry AMD is linked to the complement cascade, a part of the innate immune system. Advanced-stage AMD, i.e., geographic atrophy, can now be treated with new drugs approved for use in the US: pegcetacoplan and avacincaptad pegol. Pegcetacoplan (Syfovre, APL-2) targets C3 and avacincaptad pegol (Izervay, Zimura) targets C5, the pivotal proteins of the complement cascade. These drugs are administered by intravitreal injection. Table 2 summarizes the most important studies and information related to clinical trials conducted in dry AMD.

### 2.2. Drugs in Dry AMD

#### 2.2.1. Pegcetacoplan

Pegcetacoplan therapy regulates excessive activation of the complement cascade, a part of the immune system that can lead to the onset and progression of many diseases. The treatment was approved based on OAKS and DERBY, two multicenter randomized double-masked sham-controlled phase 3 studies comparing the efficacy and safety of intravitreal pegcetacoplan versus sham injections. The primary endpoint was the change from baseline to month 12 in the total area of geographic atrophy lesions in the study eye [20]. There have been isolated complications such as ocular and periocular infections, endophthalmitis, retinal vasculitis, retinal vascular occlusion, retinal detachment, increased intraocular pressure, and conversion to nAMD. Preliminary studies show that pegcetacoplan therapy is associated with a higher risk of vasculitis and nonarteritic ischemic optic neuropathy than avacincaptad pegol but further observations and clinical trials are needed [31].

#### 2.2.2. Avacincaptad Pegol

Avacincaptad pegol (ACP) was approved based on the GATHER1 and GATHER2 studies. ACP slowed down the growth of the GA area in the participants’ eyes compared with a sham injection. The patients who received ACP had a similar ability to read letters of different sizes on a chart one year after treatment compared with those who received no ACP [32]. In addition to the typical complications associated with the intravitreal injections, conversion to macular neovascularization was reported, alike in the case with pegcetacoplan. MNV conversion rates were 11.9% with ACP 2 mg and 15.7% with ACP 4 mg, compared to 2.7% and 2.4% in the respective sham groups. Intravitreal ACP was generally well tolerated, with no cases of endophthalmitis and only a single and mild episode of vitritis [28].

#### 2.2.3. Brimonidine Tartrate

Brimonidine, widely used in the treatment of glaucoma, has also been evaluated in dry AMD. It is a highly selective α2-adrenergic receptor agonist currently used in drop form to lower intraocular pressure in patients with glaucoma. Brimonidine also has cytoprotective and neuroprotective effects on the retina. In vitro, brimonidine decreases the production of toxic reactive oxygen species and has a protective effect on human RPE (ARPE-19) and Müller (MIO-M1) cells. α2-adrenergic receptors are expressed in RPE, neuronal cells (e.g., photoreceptors), and retinal ganglion cells and their activation by brimonidine has a cascading effect on signaling pathways that block apoptosis. Activation of the α2-adrenergic receptor increases the expression of growth factors such as basic fibroblast growth factor, inhibits the accumulation of excitotoxic levels of glutamate that cause neuronal cell death, and modifies synaptic transmission by modulating *N*-methyl-D-aspartate receptors, reducing hyperpolarization and calcium influx. So, hypothetically, brimonidine has protective effects on RPE cells and photoreceptors. An intravitreal implant containing brimonidine in a biodegradable poly(d,l-lactide) polymer matrix (Brimo DDS; Allergan, an AbbVie company, North Chicago, IL, USA) was developed as a potential GA treatment. The implant is delivered via an applicator system and slowly releases brimonidine into the vitreous over several months as the polymer matrix degrades. In the next phase of the study, to achieve faster drug release and a higher brimonidine concentration in the retina, the Brimo DDS Gen 1 was modified to Brimo DDS Gen 2, replacing brimonidine tartrate with brimonidine free base. This resulted in approximately 50% higher active drug delivery compared to the Gen 1 implant. The implant polymer was also changed to a poly(d,l-lactic acid-co-glycolic acid)/(d,l-lactic acid) blend, which biodegrades faster, resulting in accelerated brimonidine release and higher drug levels in the retina. Brimo DDS (Gen 2) appears safe and well-tolerated with repeated applications. Serious adverse events following Brimo DDS included conversion to nAMD. Other adverse events included hemorrhage into the vitreous, retinal tear, vitreous floaters, cataracts, punctate keratitis, blurred vision, and deterioration in VA. Phase 2A of the BEACON trial evaluated the outcomes of intravitreal brimonidine (delayed delivery system). A lower rate of geographic atrophy progression was demonstrated but the results did not reach statistical significance. Phase 2B showed a reduction in the progression of geographic atrophy with higher doses of brimonidine [30]. Phase 3 clinical trials exploring Brimo DDS (IMAGINE and ENVISION) are currently planned.

### 2.3. Stem Cells

Research is currently being carried out into using stem cells to treat the dry form of AMD, with the aim of replacing some of the cells that die in the late stages of the disease, such as retinal pigment epithelial cells. Human pluripotent stem cells (hPSCs) include human embryonic stem cells (hESCs) and human induced pluripotent stem cells (hiPSCs). Stem cell treatments involve delivering new RPE cells into the subretinal space and implanting cells that generate protective factors [33]. The first human study, using human embryonic stem cell-derived retinal pigment epithelial (hESC-RPE) cells, was conducted on 13 patients with dry AMD and 13 patients with Stargardt disease (STGD1). For the first three months after cell transplantation, systemic immunosuppressive therapy was used to minimize the risk of rejection but it resulted in adverse effects in 28% of patients. No signs of hyperproliferation were observed after a 4-month follow-up. The transplants’ safety and tolerability were demonstrated in 18 patients with AMD (*n* = 9) or STGD1 (*n* = 9) during a 12- to 36-month follow-up. An increase in best-corrected VA was found in 10 patients, remained stable in 7, and deteriorated in 1 patient [34,35].

### 2.4. Other Aids for Dry AMD

In patients with dry AMD, options for improving vision include using optical aids and intraocular implants. Scharioth Macula Lens and EyeMax Mono lenses have been developed for patients with AMD and cataracts. The Scharioth Macula Lens (SML) is a bifocal phakic lens. Its peripheral zone is optically neutral, while the central zone has an area of 1.5mm in diameter with an additional power of +10.0 D. This central area does not impair the distance vision or limit the visual field. The Scharioth macular lens utilizes the near triad reflex. Due to pupil constriction when focusing on close objects, light passes through the central optical zone, doubling the magnification of the image. When the pupil is dilated and the eye is focused on a distant object, there is enough space around the central optical zone for light rays to pass through the peripheral neutral part of the lens. This creates an image of the distant object on the retina, which dominates the patient’s perception over the image created by focusing light through the additional optical zone. Due to the strong magnification of the image, SML takes advantage of the residual central vision, which enables the patient to read. However, the relatively high power of the implant’s optical zone means that sharp vision is only possible from very close distances—typically about 15 cm.

The EyeMax Mono lens is very similar to a standard intraocular lens (IOL), except for the fact that the optics use a hyperspherical design to increase the breadth of focus and image quality delivered to the macular area, with a retinal eccentricity of ≤10°, magnifying the image ×1.1 to ×1.2. One of the most advanced features of this lens is to provide high image quality with reduced blur in areas where photoreceptor cell density can still produce a VA of 6/30 Snellen or better. The EyeMax Mono lens represents a new class of soft acrylic extended macular vision IOLs. There are two versions of the EyeMax Mono: one is for implantation into the lens capsule after phacoemulsification and the other is for sulcus implantation, with a standard monofocal IOL lens already in place. The EyeMax Mono can be implanted bilaterally; a hypermetropic refractive target can be selected to generate 10% to 20% image magnification with spectacle correction in severe dry AMD [36]. 

The smaller-incision new-generation implantable miniature telescope (SING IMT) is indicated for patients with stable vision impairment caused by bilateral central scotomas in the late stages of AMD. The telescope implant uses micro-optical technology to magnify images typically seen in the “straight ahead” or central visual field. The images are projected onto the healthy part of the retina that is not affected, allowing patients to see straight ahead. At present, the telescope is indicated for monocular implantation. The unimplanted eye retains peripheral vision essential for balance and orientation [37].

The IOL-Vip system consists of a high-negative power lens (−64 D) implanted in the lens capsule and a high-positive power lens (+53 D) implanted in the anterior chamber. It ensures a magnification of 1.3 without significantly compromising the peripheral visual field. Improvements in vision can be achieved when the IOL-Vip system is combined with a rehabilitation program. In particular, improvements in patients’ orientation and ability to participate in daily activities have been observed [38].

However, magnifying intraocular lens systems, such as the IOL VIP System and SING IMT, have not been widely used. The implantation procedure requires a large incision and the operation itself is technically challenging and can only be performed during cataract surgery. In addition, there is a risk of impaired distance vision and reduced visual field in the patient. The limited magnifying power of an intraocular system using Galileo’s telescope principles, its high cost, and the problematic reversibility of the procedure are also important reasons for the restricted use of these systems.

Protecting the eyes from the sun is an essential aspect of AMD prevention. Wearing sunglasses with UV filters protects the eyes from harmful rays that contribute to retinal damage and exacerbation in AMD patients.

## 3. Treatment of Neovascular AMD

nAMD is one of the leading causes of vision loss worldwide.

### 3.1. Photodynamic Therapy

Before the advent of anti-VEGF, the leading therapy for nAMD patients was photodynamic therapy (PDT), using laser energy to activate verteporfin (a photosensitizer), but it is now rarely used.

Verteporfin is a benzoporphyrin derivative with a half-life of approximately 5 h. It is a 1:1 mixture of equally active isomers BPD-MAC and BPD-MAD. Verteporfin is also known as benzoporphyrin derivative monoacid ring A or BPD-MA. This medication is a second-generation photosensitizer used in the treatment of subfoveal classic CNV secondary to AMD. When administered at the recommended dose, verteporfin is not cytotoxic. It efficiently absorbs light at a wavelength of 689 nm, while the absorption peak for protoporphyrin (a first-generation photosensitizer) is 630 nm. This allows for an increase in tissue penetration of light by approximately 50%. In addition, the relatively short half-life of verteporfin allows its rapid removal from the body, minimizing the patient’s hypersensitivity to light, usually occurring 1–2 days after PDT [39]. PDT was one of the first reasonably effective treatments for nAMD. In patients with CNV, it probably prevented vision loss, although there are doubts regarding the magnitude of this effect [40].

The essence of photodynamic therapy is a phototoxic reaction triggered by the interaction of a photosensitizing substance (verteporfin) with light of an appropriate wavelength. The method uses a diode laser producing non-thermal red light (689 nm ± 3 nm). The procedure begins with intravenous administration of verteporfin (dose of 6 mg/m^2^ in 30 mL over 10 min). The drug is activated 15 min after administration starts using a non-thermal laser light delivered to the eye under topical anesthesia. At the recommended light intensity of 600 mW/cm^2^, the required light dose of 50 J/cm^2^ is delivered in 83 s. The light passes through a fiber-optic device mounted in a slit lamp and a suitable contact lens. The greatest linear dimension (GLD) of a subretinal neovascularization lesion is determined by fluorescein angiography and fundus photography. The treatment spot size should be 1000 microns larger than the lesion’s GLD to ensure complete coverage of the lesion with a 500-micron border. To avoid damage to the optic nerve, it is recommended that the PDT laser spot does not extend within 200 microns of the optic nerve head border. As a result of the phototoxic reaction, when verteporphins are activated by light in the presence of oxygen, cytotoxic compounds are formed. When the energy absorbed by the porphyrin is transferred to oxygen, an unstable highly reactive oxygen molecule is formed. It causes damage to biological structures in the area into which it can diffuse, leading to local vascular closure, cell damage, and, under certain conditions, cell apoptosis. The selectivity of verteporfin PDT is based on localized light exposure and the selective and rapid uptake and retention of verteporfin by rapidly dividing cells, including endothelial cells of newly formed pathological choroidal vessels [39].

In the studies Verteporfin in Photodynamic Therapy (VIP) 2001 and Treatment of Age-related Macular Degeneration with Photodynamic Therapy (TAP) 1999, verteporfin was administered according to the product characteristics. Approximately five treatments were required to stabilize the clinical condition (Miller 1999; Schmidt-Erfurth 1999). In the subgroup with latent CNV, 3.1 treatments were given in the study group and 3.5 in the control group. In the second year, 1.8 and 2.4 doses were given in the verteporfin and control groups, respectively [40].

In clinical trials, the maximum treatment focus size in the first treatment cycle was 6400 µm. When treating lesions larger than this size, the light should be applied to the largest possible area of the active lesion. Follow-up examinations should be performed every 3 months. Verteporfin treatment can be repeated up to four times a year in the case of recurrence.

After treatment with verteporfin, visual disturbances such as haze, blurred vision, reduced VA, and even significant visual field loss, gloom, and dark spots may occur. Photosensitivity reactions have occurred in the form of sunburn following exposure to sunlight, usually within 24 h of verteporfin administration. To prevent this complication, patients should, within 48 h after verteporfin administration, avoid exposure of unprotected skin, eyes, or other parts of the body to sunlight or intense indoor lighting such as tanning beds, bright halogen lighting, or high-powered lighting used in operating theatres or dental surgeries. If patients need to be outdoors in daylight during the first 48 h after treatment, they must wear suitable clothing and dark glasses to protect their skin and eyes [39].

In patients with classic and latent CNV due to nAMD, photodynamic therapy is likely to prevent visual loss, although the magnitude of the effect remains questionable. In the era of more effective and safer anti-VEGF drugs, PDT is receding into the background.

### 3.2. Anti-VEGF Drugs

#### 3.2.1. General Information

**Figure 6:**
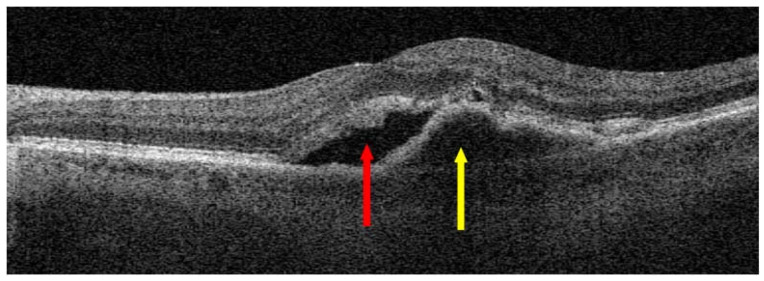
Neovascular age-related macular degeneration (nAMD) before anti-vascular endothelial growth factor (anti-VEGF) injections; red arrow points to SRF and yellow arrow shows macular neovascular membranes (MNV).

**Figure 7:**
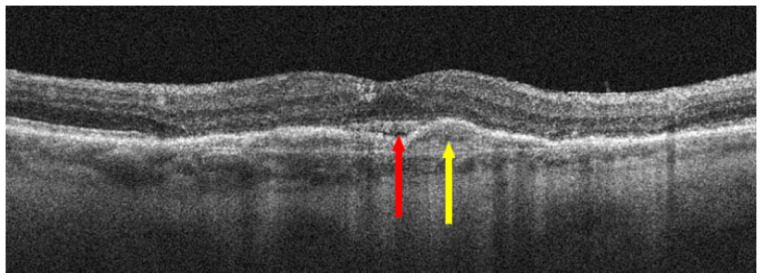
nAMD after three anti-VEGF injections; red arrow points to SRF reduction and yellow arrow shows a reduction in MNV.

**Figure 8:**
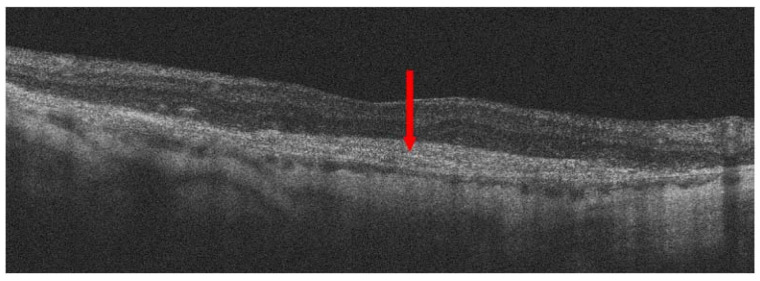
Advanced nAMD; red arrow shows a disciform scar.

**Figure 9:**
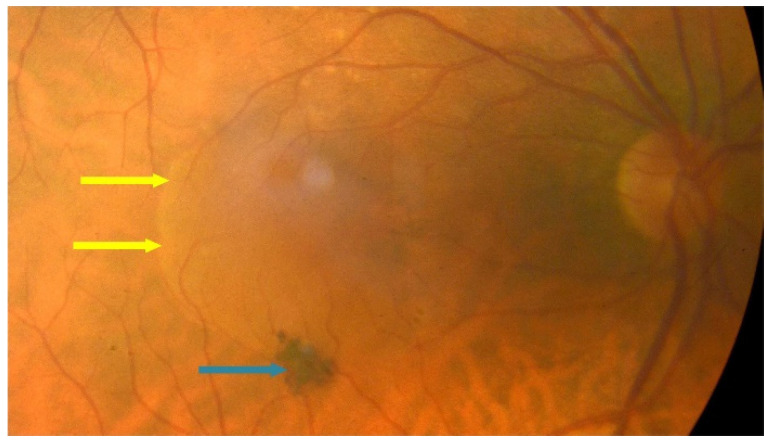
nAMD–fundus, yellow arrows show PED and blue arrow shows migration of retinal pigment epithelium (RPE) cells.

**Figure 10:**
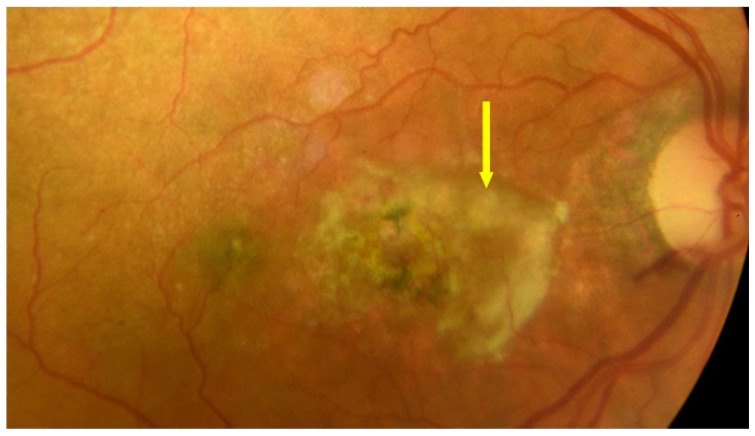
End-stage nAMD fundus; yellow arrow shows scarring.

**Figure 11:**
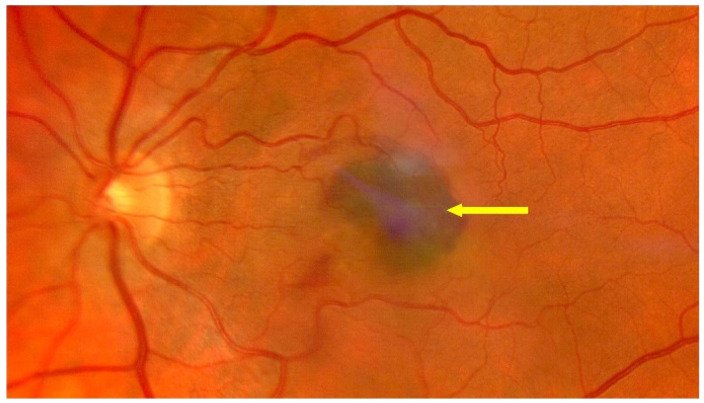
End-stage nAMD fundus; yellow arrow shows subretinal hemorrhage.

For more than 18 years, nAMD has been treated with intravitreal injections of anti-VEGFs, which are effective first-line treatments for this disease entity. This treatment has, for the first time, offered hope for slowing the progression of the disease to functional blindness and, in some cases, maintaining or even improving VA. Treatment with anti-VEGFs targets one of the critical mechanisms of nAMD development. Despite their effectiveness, anti-VEGF drugs have limitations, including the need for repeated injections with short intervals in between, the need for long-term use, and a decrease in VA associated with progressive geographic atrophy and subretinal fibrotic processes that occur with long-term treatment [41]. Successful treatment of nAMD requires a short time interval between diagnosis and the first injection of an anti-VEGF. Preferably, treatment should start as early as possible, within two weeks after diagnosis. A delay of over one month increases the risk of vision loss [42]. The frequency of intravitreal anti-VEGF injections can be reduced by using different treatment regimens, such as treat and extend, in which the intervals between anti-VEGF injections can be as long as 12 to 16 weeks [41]. Figure 6 shows an OCT scan of active MNV in a patient awaiting intravitreal anti-VEGF injections; Figure 7 shows a post-treatment OCT scan with MNV reduction and residual SRF. Figure 8 shows advanced nAMD, i.e., subretinal fibrosis and disciform scar. Figure 9, Figure 10 and Figure 11 present eye fundus in patients at various nAMD stages. Figure 9 shows an active nAMD process with a giant PED. Figure 10 shows advanced nAMD with a fundus scar: intravitreal injections are no longer administered at this stage, the changes are irreversible, and vision is unlikely to improve. Figure 11 shows hemorrhagic nAMD that certainly requires a prompt intravitreal injection of the anti-VEGF drug.

A number of anti-VEGF injection regimens are currently in use. A fixed regimen involves injections administered at specific intervals, regardless of disease activity. Regimens based on monthly injections were among the first to be analyzed in clinical trials, such as the ranibizumab MARINA and ANCHOR registration trials and aflibercept VIEW 1 and VIEW 2. These regimens achieved high efficacy after the first year of treatment. Still, they were associated with a high treatment burden and side effects, such as endophthalmitis, increased intraocular pressure, and pigment epithelial atrophy. Systemic side effects, which should also be kept in mind, are associated with reduced levels of VEGF in the blood and include hemorrhages and thromboembolic disorders.

The flexible regimen (i.e., if necessary, pro-re-nata—PRN) involves giving injections according to the current clinical condition, i.e., when there are signs of MNV activity. The disadvantage of the PRN regimen is the likelihood of overlooking the need for injections at infrequent follow-up visits. What matters is not just the number of injections given but the right timing.

The treat-and-extend regimen (i.e., treat and extend intervals between T and E/TAE doses) involves maintaining the macula without neovascularization and avoiding recurrences while reducing the number of injections and follow-up appointments. Injections are administered monthly until the intra- or SRF is absorbed and then the intervals between injections are extended by no more than two weeks. Injections are performed at each follow-up; the intervals are individually determined and shortened in case of recurrence [43].

#### 3.2.2. Anti-VEGF Therapies

**Figure 12:**
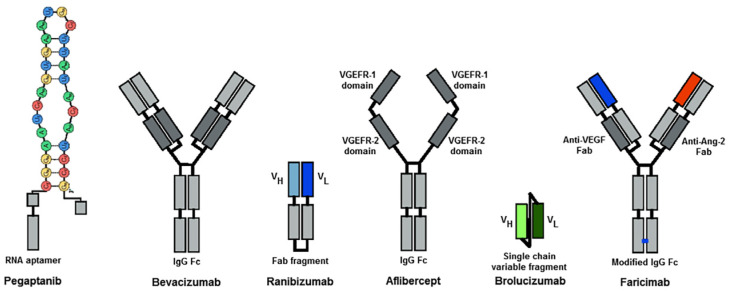
Molecular structure of anti-VEGF agents; V_L_: light chain variable domain, V_H_: heavy chain variable domain, Fab: fragment antigen binding, VEGFR: vascular endothelial growth factor receptor, IgG: immunoglobulin G, Fc: fragment crystallizable, VEGF: vascular endothelial growth factor, Ang-2: angiopoietin-2 [44].

Although intravitreal injections of anti-VEGF drugs are still the gold standard treatment for AMD, new molecules with more potent effects are being introduced. Treatment regimens are being implemented to extend the intervals between intravitreal injections. Pegaptanib was the first to be registered for the treatment of nAMD in 2004 but its effectiveness was at the level of PDT. Bevacizumab, an oncology drug with systemic VEGF-binding properties, has been used off-label to treat nAMD since 2005. There are numerous controversies related to the effectiveness and safety of nAMD treatment with bevacizumab. In 2006, ranibizumab was registered, the first approved anti-VEGF therapy that has dramatically reduced the risk of blindness worldwide. Another anti-VEGF molecule that was approved in 2012 is aflibercept, which shows high effectiveness in the treatment of nAMD and the safety of the therapies. Brolucizumab was registered in 2020. It shows good drug penetration into subretinal structures and high treatment effectiveness; however, clinical trials have shown that it may cause vasculitis, endophthalmitis, and vascular occlusion. A novel anti-VEGF drug, registered in 2022, for the treatment of nAMD is faricimab, the first bispecific antibody designed for intraocular application. It binds and neutralizes both Ang-2 and VEGF-A, thus affecting two different receptor pathways. The schematic structure of anti-VEGF molecules is presented in Figure 12 and individual anti-VEGF drugs are discussed.

The first anti-VEGF drug, pegaptanib, is no longer used. Currently, approved anti-VEGF drugs include ranibizumab, aflibercept, and brolucizumab; faricimab and bevacizumab are used as off-label drugs. Table 3 presents the most important studies on anti-VEGF drugs and information related to clinical trials in nAMD.

##### Pegaptanib

Pegaptanib sodium was the first approved intravitreal drug for the treatment of nAMD. It is a pegylated aptamer, composed of a single strand of ribonucleic acid (RNA), the structure of which enables binding and blocking VEGF 165, which plays a key role in angiogenesis and in increasing blood vessel permeability [61]. In the VISION-1 trial, pegaptanib provided statistically and clinically significant benefits in treating nAMD. For all doses, a reduced risk of VA loss was observed as early as six weeks after starting treatment, with continued beneficial effects up to week 54. Pegaptanib treatment reduced the risk of disease progression, promoted visual stability, and resulted in more remarkable visual improvement at week 54 in a small percentage of patients than in those receiving sham injections. Initially, only isoform 165 was thought to be important in nAMD. Over time, it turned out that the 121 isoform VEGF also plays a vital role in nAMD, making pegaptanib’s effectiveness comparable to PDT [62,63].

##### Bevacizumab

Bevacizumab is a recombinant humanized monoclonal antibody produced using DNA technology in Chinese hamster ovary cells. It is a full-length monoclonal antibody directed against all VEGFA isoforms. Its molecular weight is 149 kDa. Bevacizumab is administered via intravitreal at a dose of 1.25 mg. Since 2005, it has been administered off-label in the treatment of nAMD. Due to the lower cost of treatment, it is still widely used to treat nAMD, despite newer anti-VEGF drugs being approved. Pharmacodynamics (PDs) studies have shown that the binding site of bevacizumab has a 14 times smaller binding affinity for VEGF-A than ranibizumab. Intravitreal bevacizumab works according to a two-compartment model in non-vitrectomized human eyes, with initial and terminal half-lives of 0.5 and 6.7 days, respectively. The maximum concentration, 165 µg/mL, is reached on the second day after intravitreal administration [64]. Another study showed that the clearance of intravitreal bevacizumab was between 2.5 and 7.3 days, with a mean of 4.9 days. The mean half-life of intravitreally injected bevacizumab was 0.66 days in previously vitrectomized eyes [65], i.e., much shorter than in non-vitrectomized patients. It has been hypothesized that the vitreous humor acts as a reservoir of bevacizumab and that its deficiency leads to faster elimination of the drug from the eye. Avery et al. [66] showed a systemic half-life of 18.7 days after three monthly intravitreal injections. It was found that the systemic exposure of bevacizumab was greater than that of ranibizumab or aflibercept and the mean serum concentration was 1.58 nM, i.e., higher than the estimated inhibitory concentration of VEGF (IC50 = 0.668 nM). Hence, there is an increased risk of systemic adverse events (mainly cardiovascular) compared to other anti-VEGF drugs tested [67].

The ABC study was the first prospective double-masked multicenter randomized controlled trial to investigate the safety and efficacy of bevacizumab for nAMD. Bevacizumab proved more effective than intravitreal pegaptanib, verteporfin, and sham injections, with a low rate of severe ocular side effects. The treatment improved VA after an average of 54 weeks [46,68].

The CATT trial, published in 2011, compared the efficacy of ranibizumab and bevacizumab. Monthly injections improved VA and ranibizumab was more effective in reducing retinal fluid. A higher rate of serious adverse events was noted for bevacizumab. Nevertheless, CATT concluded that a favorable VA could be achieved by persistent administration of bevacizumab in patients with nAMD [69].

##### Ranibizumab

Ranibizumab has been used to treat nAMD since 2007. It is an antigen-binding Fab fragment without an Fc domain, a recombinant humanized monoclonal antibody produced in Escherichia coli cells using recombinant DNA technology, making it approximately one-third the size of bevacizumab. Ranibizumab binds with high affinity to human VEGF-A isoforms (VEGF110, VEGF112, and VEGF165). Thus, it prevents VEGF-A from binding to its receptors, VEGFR-1 and VEGFR2, inhibiting endothelial cell proliferation and new vessel formation [70]. Based on numerous clinical studies, a population pharmacokinetics (PKs) model was developed to illustrate the PKs of ranibizumab in patients with AMD. Systemic concentration–time profiles of ranibizumab were precisely determined using a single-compartment model with first-order absorption into general circulation and first-order elimination from the systemic circulation [71,72]. The vitreous elimination half-life was estimated to be approximately 9 days, while the systemic elimination half-life was approximately 2 h. Direct measurement of half-life in the aqueous humor indicates that the vitreous half-life of ranibizumab after a single intravitreal injection at a dose of 0.5 mg was 7.2 days [73]. Data on ranibizumab concentrations in vitreous samples are very limited due to the difficulty of obtaining in vivo samples from patients who do not require vitrectomy. A study by Avery et al. showed differences in systemic PKs and PDs between the three main intravitreal anti-VEGF drugs in a population of patients with nAMD. Bevacizumab and aflibercept caused rapid suppression of plasma free-VEGF after a single intravitreal injection, while plasma levels of free-VEGF remained largely unchanged in patients receiving ranibizumab. Additionally, compared to ranibizumab, bevacizumab and aflibercept cumulated in the blood after the third dose. These data may translate into systemic adverse events of topically applied anti-VEGF drugs [67].

Ranibizumab also reduces vessel permeability. In the case of MNV, intravitreal ranibizumab decreases the risk of VA loss in patients with nAMD. It even increases the chance of improvement compared to no treatment or photodynamic therapy. Numerous studies have shown that it is optimal to start treatment by giving an intravitreal injection of ranibizumab every month for at least three months at a dose of 0.5 mg, as this is when the most significant increase in BCVA was observed in most patients. Further treatment regimens should be individualized and depend on the change in BCVA, fundus examination, and OCT or optical coherence tomography or optical coherence tomography angiography (OCT-A) imaging.

Regarding the safety profile, ranibizumab was well tolerated in clinical trials [42]. The most commonly reported adverse events following injection are ocular pain, ocular congestion, increased intraocular pressure, inflammation of the vitreous or its detachment, visual disturbances, myodesopsia, conjunctival hemorrhage, ocular irritation, foreign body sensation, excessive tearing, blepharitis, dry eye, and ocular pruritus. Less commonly, severe adverse events such as endophthalmitis, blindness, retinal detachment, retinal tear, and iatrogenic post-traumatic cataracts have been observed. The efficacy of ranibizumab in the treatment of nAMD was proven in the MARINA (Minimally Classic/Occult Trial of the Anti-VEGF Antibody Ranibizumab in the Treatment of Neovascular Age-Related Macular Degeneration) and ANCHOR (Anti-VEGF Antibody for the Treatment of Predominantly Classic Choroidal Neovascularization in Age-related Macular Degeneration) studies. 

In the multicenter MARINA study, 24 intravitreal ranibizumab injections at a dose of either 0.3 mg or 0.5 mg or a sham injection were administered monthly to patients with classic or occult CNV. The treatment yielded clinically and statistically significant benefits regarding VA and angiographic lesions. Intravitreal administration of ranibizumab for two years prevented vision loss and even improved VA. There was a low proportion of severe ophthalmic adverse events [48].

The ANCHOR study compared patients with nAMD treated with 0.3 or 0.5 mg of intravitreal ranibizumab or verteporfin PDT. The advantage of ranibizumab over PDT was significant [74]. A major drawback of the MARINA and ANCHOR studies was the mandatory monthly drug administration regardless of the clinical presentation throughout the treatment period. This dosing frequency is inevitably associated with an increased risk of complications of the injection itself, as well as high treatment costs [4]. The PIER study attempted to determine whether ranibizumab should be administered monthly; the frequency of injections was reduced threefold. After the first three monthly injections, subsequent injections were given quarterly. The results were less satisfactory than those of the MARINA and ANCHOR studies. Ranibizumab halted the growth of CNV and reduced CNV leakage but the treatment efficacy decreased during quarterly dosing [4,75].

The PrONTO study yielded results comparable to the MARINA and ANCHOR studies. After an initial three-month saturation phase, the criteria for giving subsequent anti-VEGF injections were loss of 5 or more letters according to the ETDRS charts, with the co-occurrence of macular edema on OCT, an increase in retinal thickness of at least 100 µm, new macular hemorrhage, a new area of classic CNV on fluorescein angiography, and the presence of fluid persisting for at least one month after the previous injection. The PrONTO used a variable dosing regimen of intravitreal ranibizumab under OCT guidance; VA outcomes were comparable to phase 3 clinical trials, with fewer injections into the vitreous [51].

The SUSTAIN trial consisted of three initial monthly injections of ranibizumab 0.3 mg, followed by continued treatment in a pro-re-nata regimen for nine months. After the drug had been approved in Europe, patients continued treatment with 0.5 mg of ranibizumab. Based on BCVA and optical coherence tomography assessment, VA in patients in the SUSTAIN trial peaked after the first three monthly injections and declined slightly during individualized PRN therapy over the next 2 to 3 months; afterward, it remained stable throughout the treatment period [76].

Despite the encouraging results of clinical trials, real-world research has faced challenges in reproducing vision improvements. Monthly administration of ranibizumab allows for significant VA improvement but it also puts a substantial burden on patients, caregivers, clinicians, and healthcare systems.

##### Aflibercept

Aflibercept, used in ophthalmology since 2012, is produced using recombinant DNA technology and is a glycoprotein dimer with a molecular weight of 115 kDa. Aflibercept binds to circulating VEGF, “traps” it, and inhibits the activity of VEGF-A and VEGF-B, as well as PLGF, suppressing the growth of new blood vessels [70].

Aflibercept is a fully human protein consisting of VEGFR1 and VEGFR2 extracellular domains 2 and 3, fused to the Fc region of human IgG1. Aflibercept was designed by combining the amino acid sequences of the core binding domains of two human VEGF receptors into the human IgG-1 Fc framework region. Aflibercept has a high affinity for VEGF A, B, and PLGF. The three-dimensional structure of aflibercept allows simultaneous binding to both sides of the VEGF dimer. This results in a greater binding affinity of VEGF165 (kD = 0.45 pM) compared to ranibizumab (kD = 46–172 pM) and bevacizumab (kD = 58–1100 pM). Data on the pharmacokinetics of aflibercept are scarce and mainly related to animal models. It was shown that 83 days after intravitreal injection of 2 mg of aflibercept, its VEGF binding activity was comparable to the activity of ranibizumab after 30 days, which suggests a longer duration of action [77]. The offers of another study based on a population model estimated that the vitreous half-life of aflibercept in human eyes was 7.13 days [78]. In patients with nAMD, the mean maximum concentration (Cmax) of unbound aflibercept in the aqueous humor is 122 mg/L. The mean ocular half-life of free aflibercept is 9.1 days. Plasma concentrations of free aflibercept were low and transient, reaching undetectable levels in the first week after injection [79].

Aflibercept’s efficacy in treating nAMD was confirmed in Phase 3 clinical trials–VIEW 1 (2007–2011) and VIEW 2 (2008–2011). After two years of nAMD treatment to prevent BCVA loss, aflibercept proved as effective as ranibizumab [80].

PULSAR is a phase 3 randomized three-group double-masked non-inferiority 96-week trial. Patients with nAMD were randomized 1:1:1 to aflibercept 8 mg every 12 weeks, aflibercept 8 mg every 16 weeks, or aflibercept 2 mg every 8 weeks, following three initial monthly doses in all groups. Aflibercept at an 8 mg dose demonstrated efficacy and safety with extended dose intervals, potentially improving the management of patients with nAMD [55].

##### Brolcizumab

Brolucizumab is a humanized monoclonal single-chain antibody fragment (scFv) produced by recombinant DNA synthesis in *E*. *coli* cytoplasm. It acts as a VEGF inhibitor directed against all VEGF-A isoforms. The low molecular weight of 26 kDa and high solubility allows for the delivery of increased molar equivalents compared to other anti-VEGF agents, which may allow extended injection intervals. No fragment is capable of crystallization, and its small size enables better bioavailability with increased penetration from the vitreous into the subretinal space and a longer-lasting effect than full-size antibodies. Due to the small size of the molecule, this drug can be concentrated in a smaller volume, allowing for the delivery of 6 mg of brolucizumab, which is 11 times more than aflibercept, in just 50 µL. The studies reported that brolucizumab was more effective in treating nAMD in terms of fluid resolution subretinal and intraretinal than aflibercept. Following intravitreal administration, systemic concentrations were low but determinable in most patients for up to four weeks after injection. Peak serum concentrations were low and were generally observed within the first day after intravitreal injection (i.e., 6 or 24 h after injection). The half-life is 4.5–5.1 days. PK data suggest low systemic exposure following intravitreal administration of brolucizumab in patients with nAMD [67,81].

The safety and efficacy of brolucizumab for nAMD were compared to those of aflibercept in two multicenter double-masked phase 3 trials: HAWK and HARRIER. In both trials, the brolucizumab regimen of every 12 weeks or every 8 weeks was not inferior to 2 mg of aflibercept administered every 8 weeks regarding BCVA change from the baseline [56].

##### Faricimab

Faricimab, with a dual mechanism of action, is the first bispecific monoclonal intraocular antibody that binds selectively to VEGF-A and angiopoietin-2 (Ang-2), thereby affecting two distinct receptor pathways. Neutralizing Ang-2 restores blood vessel stability by reducing permeability and neovascularization and inhibiting inflammatory processes. Based on population pharmacokinetics, the maximum concentration of free (unbound to VEGF-A and Ang-2) faricimab in the plasma (Cmax) is estimated to occur approximately two days after administration. The mean (±SD) Cmax in plasma is estimated to be 0.23 (0.07) µg/mL and 0.22 (0.07) µg/mL. After repeated intravitreal administration, the mean plasma concentrations of faricimab are expected to be 0.002–0.003 µg/mL with injections every 8 weeks. Desideri et al. demonstrated dose-proportional pharmacokinetics (based on Cmax and AUC) of faricimab at a dose range of 0.5–6 mg. After monthly intravitreal injections, no accumulation of faricimab was observed in the vitreous humor or in the plasma [82]. Maximum concentrations of free faricimab in the plasma are estimated to be approximately 600 and 6000 times lower than those in the aqueous humor and body, respectively. Therefore, systemic pharmacodynamic effects are unlikely, further supported by the lack of significant changes in plasma free-VEGF and Ang-2 concentrations during faricimab treatment in clinical trials [82,83].

The TENAYA and LUCERNE trials evaluated patients randomly allocated to receive faricimab or aflibercept. In the faricimab arm, the treatment started with 6 mg of faricimab at weeks 1, 4, 8, and 12 and continued at up to every 16-week intervals depending on disease activity assessment at weeks 20 and 24. Patients randomized to the aflibercept arm received intravitreal aflibercept 2.0 mg every 4 weeks for 3 monthly initial doses and then continued on an every-8-week regimen. Faricimab was well tolerated; ocular side effects occurred with similar frequency in the faricimab and aflibercept arms and were typical of intravitreal injections. The incidence of severe ocular adverse events was also comparable. The results obtained in the TENAYA and LUCERNE trials indicate that, in patients with nAMD, intravitreal faricimab was no less effective than aflibercept in terms of improving BCVA and retinal anatomic parameters [59]. Treatment with both preparations was well tolerated. The favorable effect of faricimab on BCVA allows the treatment intervals to be extended for up to 16 weeks with efficacy comparable to aflibercept administered every eight weeks.

Intravitreal injections are generally safe but complications and side effects do occur, such as endophthalmitis, retinal tear or detachment, choroidal detachment, cataract development, a sudden increase in intraocular pressure, hemorrhage into the vitreous, and subconjunctival hemorrhage. Occlusive retinal vasculitis is a complication reported after brolucizumab, which is reported more frequently than after other anti-VEGFs. Endophthalmitis is a rare complication; it develops in 0.012–0.1% of patients within three days after injection. The most common pathogens are Coagulase-negative *Staphylococcus* spp. (59%) and Streptococcus viridans (15%); others include Staphylococcus aureus, Propionibacterium acnes, and Enterococcus fecalis. The main symptoms of endophthalmitis are pain, decreased vision, hypopyon, and vitritis. The first line of treatment for endophthalmitis is intravitreal antibiotics (vancomycin, ceftazidime, or amikacin). Early pars plana vitrectomy combined with vancomycin infusion has been suggested. Antibiotic prophylaxis does not reduce the risk of endophthalmitis; it has been found that it might contribute to a greater incidence. In addition, repeated use of topical antibiotics leads to increased resistance in the conjunctival flora [84].

#### 3.2.3. Other Therapies to Treat nAMD

##### Umedaptanib Pegol

Given the limitations of standard nAMD treatments, therapies targeting alternative mechanisms of action may prove useful. *Umedaptanib pegol* is a new-generation oligonucleotide-based *aptamer* against fibroblast growth factor 2 (anti-FGF2). The TOFU study showed that umedaptanib pegol alone or combined with aflibercept did not improve the BCVA and central subfield thickness (CST) over aflibercept alone. However, the change in BCVA and CST at the primary endpoint was negligible, suggesting that umedaptanib pegol effectively prevents disease progression. The RAMEN study confirmed the cessation of AMD progression. The TEMPURA trial pertained to previously untreated patients with nAMD. These results confirm, for the first time, the clinical validity of nAMD therapy based on anti-FGF2 aptamers [60]. Injection into the vitreous body was safe and well-tolerated. No clinically significant adverse effects were observed. Only mild iritis was described [85].

##### Hydrogels

The most common nAMD therapy, anti-VEGF drug delivery, requires monthly/bimonthly intravitreal injections. This is mainly due to the short half-life of these drugs. It can cause adverse events, including endophthalmitis, uveitis, increased intraocular pressure, and retinal detachment. Therefore, biodegradable sustained-release drug delivery systems (DDS), such as hydrogels with three-dimensional polymer networks characterized by high biocompatibility and efficacy, are constantly being developed.

Liu et al. (2019) showed that the controlled and prolonged release of ranibizumab could be achieved using degradable microsphere and hydrogel drug delivery systems developed by suspending ranibizumab-loaded poly(lactic-co-glycolic acid) microspheres within a poly(ethylene glycol)-*co*-(L-lactic-acid) diacrylate/*N*-isopropylacrylamide (PEG-PLLA-DA/NIPAAm) hydrogel. The controlled release time of ranibizumab for the tested DDS formulations was 6 months [86].

Liu et al. (2020) also evaluated the in vivo efficacy and biocompatibility of aflibercept-loaded microsphere-hydrogel DDS for a laser-induced CNV rat model. At the end of the study, an additional ∼7% reduction in CNV lesions was found in animals on aflibercept-DDS (32.69% ± 5.40%) compared to animals receiving bimonthly bolus aflibercept (25.95% ± 3.51%). An essential characteristic of biodegradable sustained-release DDS administered intravitreally is the clearance of residual polymer from the vitreous body. Aflibercept-DDS has been designed so that the microspheres degrade first, releasing the active aflibercept; the hydrogel degrades once the drug release is complete. The hydrogel’s delayed degradation is to ensure that the microspheres containing the drug are not released too early from the hydrogel complex. Aflibercept-DDS proved effective in treating CNV over 6 months; it was safe, well-tolerated, and biocompatible. These findings suggest that a single administration of aflibercept-DDS can be as equally effective as repeated bolus injections of aflibercept in treating CNVs. The results of the current in vivo biocompatibility study corresponded well with our previous in vitro study (2019), demonstrating that no cytotoxicity was caused by degraded by-products of the DDS [87].

Lee et al. developed a bevacizumab delivery system. The pre-crosslinked hydrogel implant (hydrogel rods) was designed to reduce the limitations of in situ-forming hydrogels and extend the intravitreal half-life of the anti-VEGF administered into the vitreous body, reducing the frequency of intraocular injections.

The adjustable degree of cross-linking of the hydrogel implants (rods) allows the release of bevacizumab to be controlled. Unlike in situ-forming hydrogels, hydrogel rods reduce the initial burst release and show sustained release and in vitro efficacy of the drug. As a result, the half-life of bevacizumab in the vitreous body and retina is significantly increased; drug release is extended to 4 months [88].

Yu et al. formed an in situ bevacizumab-loaded hydrogel by the catalyst-free chemical crosslinking between vinylsulfone functionalized hyaluronic acid (HA-VS) and thiolated dextran (Dex-SH) at physiological conditions. The pH 7.4 buffered mixture with HA-VS, Dex-SH, and bevacizumab was injected into rabbit vitreous through a 30-G needle. After injection, no hemorrhage, retinal detachment, inflammation, or other severe pathologies were observed. The concentration of bevacizumab 6 months after hydrogel injection was approximately 10^7^ times higher than after bolus injection. The biodegradability and safety of hydrogel implants seem to confirm their applicability as an advanced intraocular DDS for treating CNV-related retinal diseases [89].

The controllability of the number and type of particles loaded in the hydrogels can increase drug delivery, while the encapsulation of polymeric particles in hydrogels acts as an additional diffusion barrier to extend drug release time. However, it is essential to note that the migration of the hydrogel particles may cause glaucoma or ocular inflammation [86]. In addition, the pharmacokinetic profiles of anti-VEGF drugs are suboptimal, as the maximum drug concentration after injection might prove toxic. On the other hand, rapid clearance at a later time may result in subtherapeutic drug concentrations. Therefore, developing a DDS for anti-VEGF could ensure controlled drug delivery and reduce the frequency of IVT injections [87]. However, polymeric hydrogel carriers have several benefits, including biodegradability, biocompatibility, and a lack of cytotoxicity [90].

##### Intraocular Port Delivery System

Intraocular port delivery systems (PDS) include surgically implanted reservoirs that require periodic refills. These systems were developed to allow continuous delivery of anti-VEGF agents directly into the vitreous body through passive diffusion. An example is the ranibizumab port delivery system (Susvimo, Genentech), which was designed to reduce the frequency of ranibizumab injections in patients with nAMD. Susvimo should be refilled every 24 weeks; 100 mg per milliliter of ranibizumab is delivered to a 20-μL drug reservoir through a self-sealing septum. PDS body penetrates into the vitreous cavity and becomes anchored by an extrascleral flange resting beneath the conjunctiva and Tenon capsule. The US FDA approved the Susvimo PDS in 2021 but the product was eventually withdrawn from the market and is no longer available [22,91]. 

##### Gene Therapy

Gene therapy for nAMD is based on gene transformation and potentially provides long-lasting benefits with reduced injection frequency. It mainly targets the delivery of genes encoding anti-VEGF proteins. Anti-VEGF therapies have shown that genes related to the VEGFA/HIF-α signaling pathway (VEGF, VEGFR, PDGF, and PEGF) can enhance the effectiveness of treatment and should therefore be targeted by gene therapy in nAMD.

There is a pressing need for novel treatments to ensure sustained VEGF-A suppression. Gene products are designed to inhibit VEGF, similar to injectable anti-VEGF agents, but with a potentially extended therapeutic effect.

Breakthrough technologies such as single-cell mRNA sequencing (scRNA-Seq) and genome-wide association studies (GWAS) have revealed factors (including mutations) contributing to AMD progression. GWAS have demonstrated that specific genes, including complement factor H (CFH) on chromosome 1 and ARMS2 and HTRA1 residing on chromosome 10, are significant loci closely associated with advanced AMD.4 The CHF variant is predominantly related to the presence of drusen, while the ARMS2-HTRA1 variant is correlated with the development of subretinal or sub-RPE hemorrhages. On the other hand, MMP9, CETP, and TIMP3 have been linked to AMD due to their role in regulating extracellular matrix remodeling. The FGD6, HTRA1, and CFH genes play a crucial role in controlling oxidative stress and inflammation and, by influencing processes related to angiogenesis, contribute to the progression of nAMD [22].

To ensure the efficacy and safety of gene therapy, it is crucial to use a vector that provides prolonged levels of gene expression while minimizing the risk of toxicity and immune reactions.

Viral vectors are modified viruses commonly used in gene therapy to deliver therapeutic genes or RNA-based molecules. They have been used as carriers to precisely transport therapeutic genetic material to target cells in the eye and elicit a durable therapeutic effect.

The optimal vector for nAMD therapy is the recombinant AAV, a small single-stranded DNA genome of approximately 4.6 kilobases (kb) in size that promotes genetic modification. AAV provides numerous advantages, such as prolonged transgene expression, minimal risk of insertional mutagenesis, little inflammatory response, and a low risk of transmission to the germline.

Retroviral and lentiviral vectors are also effective in treating nAMD. Subretinal administration of a lentiviral vector expressing endostatin and angiostatin was effective and well tolerated. Patients with severe nAMD showed clinical improvement, including stabilization of VA and reduction in vascular leakage. However, retroviruses and lentiviruses carry risks, such as a chance of insertional mutagenesis and transmission to the germline. In addition, they may induce a more pronounced inflammatory response than AAV.

Subretinal injection is the predominant delivery method in gene therapy trials targeting monogenic diseases. It consists of performing a retinotomy near the temporal vascular arcades, allowing the bleb to spread slowly toward the fovea, creating a shallow elevation. Although involving a temporary retinal detachment, the method is generally safe and effective [22].

Phase 2 clinical trials of the RGX-314 gene therapy (currently referred to as ABBV-RGX-314) use an adeno-associated serotype 8 vector expressing an anti-VEGF-A antigen-binding fragment, providing potential for continuous suppression of VEGF-A after a single subretinal injection.

Subretinal administration of RGX-314 was generally well tolerated, with no clinically overt autoimmune side effects. RGX-314 gene therapy is a novel approach to sustained VEGF-A suppression in patients with nAMD that can control exudation, maintain or improve VA, and reduce treatment burden (single administration) [92].

Similarly, ADVM-022, an adeno-associated virus vector encoding aflibercept, aims to provide sustained expression of this anti-VEGF following a single intravitreal injection. Most clinical trials of gene therapy to provide sustained levels of anti-VEGF in the retina have involved subretinal injections requiring vitrectomy. A single intravitreal injection of a gene therapy product could dramatically reduce the treatment burden. Intravitreal delivery of ADVM-022 was well tolerated and yielded sustained levels of aflibercept in ocular tissues. A single intravitreal injection of ADVM-022 may provide a safe and effective long-term treatment option for nAMD. Clinical trials are underway to evaluate the safety and efficacy of a single intravitreal administration of ADVM-022 [93].

Following ADVM-022 gene therapy, the main adverse effect was inflammation, mainly affecting the anterior segment. No posterior segment inflammation, vasculitis, or intraocular inflammation were reported. All treatment-related ocular adverse events were mild (80%) or moderate (20%). Mild intraocular pressure elevation occurred in two patients, resolving with anti-glaucoma eye drops. RGX-314 therapy is generally well-tolerated; there have been no reports of therapy-related endophthalmitis. However, significant deterioration in vision has been reported, probably related to high-dose drug administration. Transvitreal subretinal delivery requires pars plana vitrectomy (PPV), which may be associated with complications such as hemorrhage, cataract, endophthalmitis, and retinal detachment. AAV8 is delivered through suprachoroidal microneedles, thus enabling the transduction of multiple retinal cell types in a broad area without invasive PPV. The suprachoroidal space lies beyond the outer blood–retina barrier formed by the RPE layer, which poses a potential risk for the host’s immune response to a viral particle or transgene [94].

Delivery techniques include not only viral but also physical delivery. The latter involves the injection of naked plasmid DNA, siRNA, mRNA, or miRNA. However, these methods are of limited efficacy due to rapid substrate degradation and minimal uptake. Gene delivery by chemical techniques appears to be a more favorable form due to its lower potential to induce an immune response.

Human trials with a modified naked RNA (bevasiranib) and a chemically modified naked siRNA (AGN211745) were terminated as it was considered that the primary objective was unlikely to be attained due to RNA instability and limited bioavailability. Furthermore, due to the rapid degradation of the drug (3 to 7 days), there was still the need for repeated injections. Nevertheless, chemical modifications of siRNA or the use of viral vectors could extend the duration of the drug’s action, which could help maintain the efficacy of therapies based on RNA interference. An alternative to siRNA is to use microRNAs (miRNAs), which are small 18–22 nucleotide single-stranded non-coding RNAs. Studies have shown that miRNA dysregulation is involved in both experimental models of AMD and AMD patients and could, therefore, be associated with an increased risk of developing AMD. Substances that mimic microRNAs or anti-miRNAs could evolve into biomarkers to treat nAMD by modulating retinal cell function [22].

### 3.3. Surgical Treatment

The surgical treatment of AMD involves attempts to remove the neovascular membrane or subretinal hemorrhage, translocate the macula, and transplant pigment epithelial cells and stem cells. Surgical removal of subretinal neovascular membranes as a treatment for AMD has been used as an alternative to laser treatment. Subretinal surgery requires PPV and retinotomy to access the subretinal space. The neovascular membrane, scar tissue, and possibly subretinal hemorrhage are removed. Macular translocation is another surgical intervention for AMD; it can be performed after partial or complete displacement of the retina with 360 retinotomy. Numerous complications are observed after this surgery, including retinal detachments and tears, macular holes, macular wrinkling, and intraocular hemorrhage.

## 4. Summary

AMD is a complex eye disease influenced by many genetic, molecular, and behavioral factors contributing to its pathogenesis and development. There are many potential therapies for AMD. New treatment options for dry AMD include arresting the progression of the disease or restoring and stabilizing retinal cells. The gold standard of treatment is currently intravitreal injections of anti-VEGF drugs. The latest research is diverse and dynamic, suggesting that more effective options will become available to protect against vision loss due to AMD. Improvements in drug delivery are likely to evolve toward the development of new pharmacological agents and more efficient delivery of currently available drugs. High hopes have been pinned on gene therapy, which could become an alternative to intravitreal injections. Gene therapy involves gene transfer to produce endogenous angiogenic inhibitors in the eyeball. Two routes of gene vector delivery are being investigated, i.e., subretinal injection during surgery and intravitreal injection. Despite the hopes placed on gene therapy, its long-term efficacy is difficult to predict. Such treatment can also be costly and may not be appropriate for all patients suffering from AMD.

## Figures and Tables

**Table 1:** Thirty-seven neovascular age-related macular degeneration (nAMD) genes; MIM—Mendelian Inheritance in Man.

Name/Gene ID	Description	Location	Aliases	MIM
3075CFH ID: 3075	complement factor H [Homo sapiens (human)]	Chromosome 1, NC_000001.11 (196652043..196747504)	AHUS1, AMBP1, ARMD4, ARMS1L3, FH, FHL1, HF, HF1, HF2, HUS, CFH	134370
5654HTRA1 ID: 5654	HtrA serine peptidase 1 [Homo sapiens (human)]	Chromosome 10, NC_000010.11 (122461553..122514907)	ARMD7, CADASIL2, CARASIL, HtrA, L56, ORF480, PRSS11	602194
387715ARMS2 ID: 387715	age-related maculopathy susceptibility 2 [Homo sapiens (human)]	Chromosome 10, NC_000010.11 (122454653..122457352)	ARMD8	611313
7422VEGFA ID: 7422	vascular endothelial growth factor A [Homo sapiens (human)]	Chromosome 6, NC_000006.12 (43770211..43786487)	L-VEGF, MVCD1, VEGF, VPF	192240
718C3 ID: 718	complement C3 [Homo sapiens (human)]	Chromosome 19, NC_000019.10 (6677704..6720650, complement)	AHUS5, ARMD9, ASPa, C3b, CPAMD1, HEL-S-62p, C3	120700
348APOE ID: 348	apolipoprotein E [Homo sapiens (human)]	Chromosome 19, NC_000019.10 (44905796..44909393)	AD2, APO-E, ApoE4, LDLCQ5, LPG	107741
1401CRP ID: 1401	C-reactive protein [Homo sapiens (human)]	Chromosome 1, NC_000001.11 (159712289..159714589, complement)	PTX1	123260
5176SERPINF1 ID: 5176	serpin family F member 1 [Homo sapiens (human)]	Chromosome 17, NC_000017.11 (1762060..1777565)	EPC-1, OI12, OI6, PEDF, PIG35	172860
1524CX3CR1 ID: 1524	C-X3-C motif chemokine receptor 1 [Homo sapiens (human)]	Chromosome 3, NC_000003.12 (39263494..39292966, complement)	CCRL1, CMKBRL1, CMKDR1, GPR13, GPRV28, V28	601470
3791KDR ID: 3791	kinase insert domain receptor [Homo sapiens (human)]	Chromosome 4, NC_000004.12 (55078481..55125595, complement)	CD309, FLK1, VEGFR, VEGFR2	191306
3576CXCL8 ID: 3576	C-X-C motif chemokine ligand 8 [Homo sapiens (human)]	Chromosome 4, NC_000004.12 (73740569..73743716)	GCP-1, GCP1, IL8, LECT, LUCT, LYNAP, MDNCF, MONAP, NAF, NAP-1, NAP1, SCYB8	146930
6499SKIC2 ID: 6499	SKI2 subunit of superkiller complex [Homo sapiens (human)]	Chromosome 6, NC_000006.12 (31959175..31969751)	170A, DDX13, HLP, SKI2, SKI2W, SKIV2, SKIV2L, SKIV2L1, THES2	600478
710SERPING1 ID: 710	serpin family G member 1 [Homo sapiens (human)]	Chromosome 11, NC_000011.10 (57597685..57614848)	C1IN, C1INH, C1NH, HAE1, HAE2	606860
1295COL8A1 ID: 1295	collagen type VIII alpha 1 chain [Homo sapiens (human)]	Chromosome 3, NC_000003.12 (99638594..99799217)	C3orf7	120251
20296Ccl2 ID: 20296	C-C motif chemokine ligand 2 [Mus musculus (house mouse)]	Chromosome 11, NC_000077.7 (81926403..81928278)	HC11, JE, MCAF, MCP-1, SMC-CF, Scya2, Sigje	
CFB ID: 629	complement factor B [Homo sapiens (human)]	Chromosome 6, NC_000006.12 (31946095..31952084)	AHUS4, ARMD14, BF, BFD, CFABD, FB, FBI12, GBG, H2-Bf, PBF2, CFB	138470
CFI ID: 3426	complement factor I [Homo sapiens (human)]	Chromosome 4, NC_000004.12 (109730982..109801999, complement)	AHUS3, ARMD13, C3BINA, C3b-INA, FI, IF, KAF	217030
C2 ID: 717	complement C2 [Homo sapiens (human)]	Chromosome 6, NC_000006.12 (31897783..31945672)	ARMD14, CO2	613927
FLT1 ID: 2321	fms related receptor tyrosine kinase 1 [Homo sapiens (human)]	Chromosome 13, NC_000013.11 (28300346..28495128, complement)	FLT, FLT-1, VEGFR-1, VEGFR1	165070
CETP ID: 1071	cholesteryl ester transfer protein [Homo sapiens (human)]	Chromosome 16, NC_000016.10 (56961950..56983845)	BPIFF, HDLCQ10	118470
TLR3 ID: 7098	toll like receptor 3 [Homo sapiens (human)]	Chromosome 4, NC_000004.12 (186069156..186088073)	CD283, IIAE2, IMD83	603029
CFHR3 ID: 10878	complement factor H related 3 [Homo sapiens (human)]	Chromosome 1, NC_000001.11 (196774840..196795407)	CFHL3, DOWN16, FHR-3, FHR3, HLF4	605336
Ahr ID: 11622	aryl-hydrocarbon receptor [Mus musculus (house mouse)]	Chromosome 12, NC_000078.7 (35547978..35584988, complement)	Ah, Ahhe, In, bHLHe76, Ahr	
PGF ID: 5228	placental growth factor [Homo sapiens (human)]	Chromosome 14, NC_000014.9 (74941830..74955764, complement)	D12S1900L, PIGF, PLGF, PLGF-2, SHGC-10760, PGF	601121
CD36 ID: 948	CD36 molecule (CD36 blood group) [Homo sapiens (human)]	Chromosome 7, NC_000007.14 (80602207..80679274)	BDPLT10, CHDS7, FAT, GP3B, GP4, GPIV, PASIV, SCARB3	173510
THBS1 ID: 7057	thrombospondin 1 [Homo sapiens (human)]	Chromosome 15, NC_000015.10 (39581079..39599466)	THBS, THBS-1, TSP, TSP-1, TSP1	188060
RORA ID: 6095	RAR related orphan receptor A [Homo sapiens (human)]	Chromosome 15, NC_000015.10 (60488284..61229302, complement)	IDDECA, NR1F1, ROR1, ROR2, ROR3, RORa1, RORalpha, RZR-ALPHA, RZRA	600825
TOMM40 ID: 10452	translocase of outer mitochondrial membrane 40 [Homo sapiens (human)]	Chromosome 19, NC_000019.10 (44891254..44903689)	C19orf1, D19S1177E, PER-EC1, PEREC1, TOM40	608061
CFP ID: 5199	complement factor properdin [Homo sapiens (human)]	Chromosome X, NC_000023.11 (47623282..47630305, complement)	BFD, PFC, PFD, PROPERDIN	300383
C9 ID: 735	complement C9 [Homo sapiens (human)]	Chromosome 5, NC_000005.10 (39284140..39364495, complement)	ARMD15D, C9	120940
TGFB1 ID: 7040	transforming growth factor beta 1 [Homo sapiens (human)]	Chromosome 19, NC_000019.10 (41330323..41353922, complement)	CED, DPD1, IBDIMDE, LAP, TGF-beta1, TGFB	190180
CXCL12 ID: 6387	C-X-C motif chemokine ligand 12 [Homo sapiens (human)]	Chromosome 10, NC_000010.11 (44370165..44385097, complement)	IRH, PBSF, SCYB12, SDF1, TLSF, TPAR1	600835
TIMP3 ID: 7078	TIMP metallopeptidase inhibitor 3 [Homo sapiens (human)]	Chromosome 22, NC_000022.11 (32801705..32863041)	HSMRK222, K222, K222TA2, SFD	188826
ELN ID: 2006	elastin [Homo sapiens (human)]	Chromosome 7, NC_000007.14 (74028173..74069907)	ADCL1, SVAS, WBS, WS	130160
CXCR3 ID: 2833	C-X-C motif chemokine receptor 3 [Homo sapiens (human)]	Chromosome X, NC_000023.11 (71615919..71618511, complement)	CD182, CD183, CKR-L2, CMKAR3, GPR9, IP10-R, Mig-R, MigR	300574
ACAD10 ID: 80724	acyl-CoA dehydrogenase family member 10 [Homo sapiens (human)]	Chromosome 12, NC_000012.12 (111686053..111757099)		611181
MIR4513 ID: 100616183	microRNA 4513 [Homo sapiens (human)]	Chromosome 15, NC_000015.10 (74788672..74788757, complement)		

**Table 2:** Clinical trials conducted in dry AMD.

Name of the Study	Date	Investigated Drug	Number of Participants	Results	Ocular Adverse Events
OAKS [27]	2018–2020 (24 months)	pegcetacoplan	637	% GA reduction in treated vs. sham Monthly: 21% (*p* = 0.0528)	1.6%
DERBY [27]	2018–2020 (24 months)	pegcetacoplan	621	% GA reduction in treated vs. sham Monthly: 12% (*p* = 0.0528)	1.3%
GATHER1 [28]	2016–2019 (18 months)	avacincaptad pegol	286	% GA reduction in treated vs. sham 2 mg: 27.4% (*p* = 0.0072) 4 mg: 27.8% (*p* = 0.0051)	≥2%
GATHER2 [29]	2020–2021 (12 months)	avacincaptad pegol	448	% GA reduction in treated vs. sham 2 mg:14% (*p =* 0.0064)	49%
BEACON [30]	2014–2018 (30 months)	brimonidine	310	% GA reduction in treated vs. sham 400 μg: 10% (*p* = 0.033)	62.3%

**Table 3:** Clinical trials in nAMD.

Name of the Study	Date	Investigated Drug	Number of Participants	Results	Ocular Adverse Events
VISION-1 [45]	2001–2002 54 weeks	pegaptanib	1190	In the pegaptanib 0.3 mg group, 80% achieved the primary endpoint of <15 ETDRS charts (Early Treatment Diabetic Retinopathy Study) letters lost, 47% maintained VA, and 20% gained ≥15 letters of vision	Serious ocular adverse events: 0.16% endophthalmitis and 0.08% retinal detachment
ABC [46]	2006–2007	bevacizumab	131	In the bevacizumab group, 21 (32%) patients achieved 15 or more letters above baseline VA compared with two (3%) in the standard treatment group (*p* < 0.001).	Serious ocular adverse events associated with bevacizumab were uncommon. Rates of adverse events of intraocular inflammation graded as ≥1
CATT [47]	2008–2012	bevacizumab ranibizumab	1208	VA was similar for both drugs (bevacizumab-ranibizumab difference, −1.4 letters; 95% confidence interval–CI, −3.7 to 0.8; *p* = 0.21); the mean gain was greater for monthly than for as needed treatment (difference, −2.4 letters; 95% CI, −4.8 to −0.1; *p* = 0.046)	24.1% bevacizumab 19.0% ranibizumab
MARINA [48]	2003 12 months	ranibizumab	716	VA improved by 15 or more letters in 24.8% of the 0.3-mg group and 33.8% of the 0.5-mg group, as compared with 5.0% of the sham-injection group (*p* < 0.001 for both doses)	1.3%
ANCHOR [49]	2003–2006 12 months	ranibizumab	423	VA improved 15 letters more at 12 months: 40% ranibizumab 0.5 mg, 36% Ranibizumab 0.3 mg, and 6% PDT (*p* < 0.0001)	0.3 mg ranibizumab 1.3% 0.5 mg ranibizumab 2.9%
PIER [50]	2004–2005 12 months	ranibizumab	184	Gaining at least 15 letters: 9.5% in the sham group, 11.7% ranibizumab 0.3 mg, and 13.1% ranibizumab 0.5 mg	The incidence of ocular adverse events was low. Ocular hemorrhage: sham 1.6% 0.3 mg ranibizumab 3.4% 0.5 mg ranibizumab 0% Macular edema Sham 1.6% 0.3 mg ranibizumab 1.7% 0.5 mg ranibizumab 0%
PrONTO [51]	2004–2005 24 months	ranibizumab	40	VA improved by 11.1 letters (*p* < 0.001); the OCT-CRT decreased by 212 microm (*p* < 0.001)	There were no ocular adverse events attributable to the injection of ranibizumab
SUSTAIN [52]	2006–2008 12 months	ranibizumab	531	Mean best-corrected VA increased from baseline to month 3 to reach +5.8 letters, decreased slightly from month 3 to 6, and remained stable from month 6 to 12, reaching +3.6 at month 12	Serious ocular AE: 1.2% both 0.3 mg and 0.5 mg of ranibizmab
VIEW1 [53]	2007–2011 12 months	alibercept	1217	Mean improvements from baseline in the ETDRS letter score for 0.5 mg ranibizmab 8.1 letters, 0.5 aflibercept 6.9 letters, 2 mg aflibercept every month 10.9 letters, 2 mg aflibercept every 2 months 7.9 letters, and 2 mg afibercept every month was significantly better (*p* < 0.01) than 0.5 mg ranibizumab	Serious AE: 3.3% ranibizumab and 1.0% alibercept. The most frequent ocular AEs were conjunctival hemorrhage, macular degeneration, eye pain, vitreous detachment, and vitreous floaters
VIEW2 [54]	2008–2011	aflibercept	1240	Mean improvements from baseline in ETDRS letter score for 0.5 mg ranibizmab 9.4 letters, 2 mg aflibercept every month 7.6 letters, and 2 mg aflibercept every 2 months 8.9 letters	Serious AE: 3.1% ranibizumab and 2.9% alibercept. The most frequent ocular AEs were conjunctival hemorrhage, macular degeneration, eye pain, vitreous detachment, and vitreous floaters
PULSAR [55]	24 months 2020–2021	aflibercept	1011	Aflibercept 8q12 and 8q16 showed non-inferior best-corrected visual acuity (BCVA) gains versus aflibercept 2q8. Mean BCVA change from baseline: 8q12 +6·7 (standard deviation SD 12.6 letters) 8q16 +6·2 (SD 11.7 letters 2q8 +7·6 (SD 12.2 letters).	Ocular adverse events in the study eye was similar across groups: 39% aflibercept 8q12, 38% aflibercept 8q16, and 39% aflibercept 2q8
HAWK [56,57]	24 months 2014–2017	brolucizumab	1078	Each brolucizumab arm demonstrated noninferiority to aflibercept in BCVA change from baseline, least squares LS mean: 6 mg brolucizumab +6.6 letters 3 mg broucizumab +6.1 letters 2 mg aflibercept +6.8 letters	Ocular adverse events: 2.2% brolucizumab 6 mg 0.3% brolucizumab 3 mg 0% aflibercept 2 mg Thromboembolic events: 1.1% brolucizumab 3 mg, 1.4% brolucizumab 6 mg, 0.3% aflibercept 2 mg
HARRIER [56,57]	2015–2017 24months	brolucizumab	739	Brolucizumab arm demonstrated noninferiority to aflibercept in BCVA: 6 mg brolucizumab +6.4 letters 2 mg aflibercept +3.7 letters	Ocular adverse events occurring in ≥3%, thromboembolic events: 1.6% brolucizumab 6 mg 0.5% aflibercept 2 mg
TENAYA [58,59]	2019–2022 112 weeks	faricimab	671	BCVA change from baseline with faricimab was non-inferior to aflibercept, adjusted mean change: 6 mg faricimab +3.7 letters 2 mg aflibercept +3.3 letters	36.3% faricmabvs 38.1% aflibercept
LUCERNE [58,59]	2019–2022 112 weeks	faricimab	658	BCVA change from baseline with faricimab was non-inferior to aflibercept: 6 mg faricimab 5.0 letters 2 mg aflibercept 5.2 letters	40.2% faricimab 36.2 alibercept
TOFU [60]	2019–2021 4 months	umedaptanib pegol (anti-FGF2)	86	Umedaptanib pegol alone or in combination with aflibercept did not improve BCVA, which suggests that umedaptanib pegol is effective in preventing the disease progression	Subjects with at least one Ocular a treatment-emergent adverse event (TEAE): Arm 1: 57.1% Arm 2: 65.5% Arm 3: 34.5%
RAMEN Extension TOFU Trial [60]	2020–2021 4 months	umedaptanib pegol (anti-FGF2)	22	The RAMEN study confirmed the cessation of disease progression	No drug-related adverse events were reported Ocular adverse events were related to the intravitreal injection procedure
TEMPURA [60]	2021–2022 4 months	umedaptanib pegol (anti-FGF2)	5	In the TEMPURA study, naïve nAMD patients showed improvement and no further macular degeneration	No drug-related adverse events were reported; 1 ocular adverse event was reported—1 subretinal hemorrhage—20%

## Data Availability

Not applicable.
